# Specific nuclear envelope transmembrane proteins can promote the location of chromosomes to and from the nuclear periphery

**DOI:** 10.1186/gb-2013-14-2-r14

**Published:** 2013-02-15

**Authors:** Nikolaj Zuleger, Shelagh Boyle, David A Kelly, Jose I de las Heras, Vassiliki Lazou, Nadia Korfali, Dzmitry G Batrakou, K Natalie Randles, Glenn E Morris, David J Harrison, Wendy A Bickmore, Eric C Schirmer

**Affiliations:** 1 The Wellcome Trust Centre for Cell Biology, University of Edinburgh, Mayfield Road, Edinburgh, EH9 3JR, UK; 2 MRC Human Genetics Unit, Institute of Genetics and Molecular Medicine, Crewe Road, Edinburgh, EH4 2XU, UK; 3 Wolfson Centre for Inherited Neuromuscular Disease, RJAH Orthopaedic Hospital, Twympath Lane, Oswestry, SY10 7AG, UK; 4 Institute for Science and Technology in Medicine, Keele University, Keele, Staffordshire, ST4 7QB, UK; 5 School of Medicine, Medical andBiological Sciences, University of St Andrews, North Haugh, St Andrews, KY16 9TF, UK

## Abstract

**Background:**

Different cell types have distinctive patterns of chromosome positioning in the nucleus. Although ectopic affinity-tethering of specific loci can be used to relocate chromosomes to the nuclear periphery, endogenous nuclear envelope proteins that control such a mechanism in mammalian cells have yet to be widely identified.

**Results:**

To search for such proteins, 23 nuclear envelope transmembrane proteins were screened for their ability to promote peripheral localization of human chromosomes in HT1080 fibroblasts. Five of these proteins had strong effects on chromosome 5, but individual proteins affected different subsets of chromosomes. The repositioning effects were reversible and the proteins with effects all exhibited highly tissue-restricted patterns of expression. Depletion of two nuclear envelope transmembrane proteins that were preferentially expressed in liver each reduced the normal peripheral positioning of chromosome 5 in liver cells.

**Conclusions:**

The discovery of nuclear envelope transmembrane proteins that can modulate chromosome position and have restricted patterns of expression may enable dissection of the functional relevance of tissue-specific patterns of radial chromosome positioning.

## Background

It is well established that specific chromosomes, chromosome regions, and/or chromatin domains have preferred positions in the nucleus. For example, gene-poor chromosomes/regions tend to be at the nuclear periphery while gene-rich regions locate to the interior [1-5]. This radial organization can be modulated by the physiological and pathological state of the cell [6-8]. Genome organization can also be tissue specific [9-11]. For example, mouse chromosome 5 tends to be in the nuclear interior in liver but at the periphery in lung [12]. More dramatically, the radial organization of chromatin completely inverts in the nuclei of rod photoreceptors in nocturnal mammals [13]. In addition to visual methods, molecular approaches also reveal changes in associations of the mammalian genome with the nuclear lamins upon differentiation [14].

One might easily postulate that peripheral localization would depend on connections to the nuclear envelope (NE). Indeed, many NE proteins have chromatin binding activities important for reforming the NE at the end of mitosis [15,16] and a high-affinity interaction established at this time can direct interphase chromosome positioning [17-19]. However, most NE-chromatin interactions described involve widely expressed proteins (reviewed in [20]) mediating interactions with heterochromatin [21-25]. Such interactions provide a mechanism to maintain inactive chromatin at the periphery that could explain the partial relationship between chromosome positioning and gene density. However, they do not explain how in some tissues particular chromosomes reposition to the nuclear periphery. Some work has been done indicating components that may be involved from the chromatin side, but the only proteins implicated from the NE thus far are lamins. However, though lamin B1 contributes to retention of specific chromosomes at the nuclear periphery, its ubiquitous presence is inconsistent with it directing tissue-specific chromosome positioning without additional factors [26,27]. That lamins support this process is further indicated by mutant forms of lamin A resulting in altered chromosome positioning in human nuclei [8,28].

To identify other NE factors, 22 novel NE transmembrane proteins (NETs) [29] and emerin were screened for their ability to contribute to chromosome positioning patterns in human cells. Five NETs (NET5, NET29, NET39, NET45 and NET47) promoted repositioning of chromosome 5 to the periphery, but only two of these (NET29 and NET39) did so for chromosome 13. These NETs exhibited restricted tissue expression and several were preferentially expressed in liver with, for example, NET45 and NET47 expressed roughly 20-fold higher in liver than in kidney. Correspondingly, chromosome 5 was more peripheral in the nuclei of human liver than in kidney. Chromosome repositioning effects of NETs were reversible; most notably, depletion of the two most liver-specific NETs in liver cells reduced the normal peripheral positioning of chromosome 5. Thus, we postulate that these NETs may play a role in generating the more particular patterns of chromosome organization observed in certain tissues.

## Results

### A screen for NETs that influence chromosome positioning

To identify NE proteins that contribute to genome organization, 22 novel NETs identified in a proteomic study of liver NEs [29] and the well-characterized NET emerin were screened to determine if they could influence the position relative to the nuclear periphery of lacO arrays integrated in two different human chromosomes. The inner nuclear membrane localization of most of the NETs tested here has been confirmed by super-resolution light microscopy and/or immuno-electron microscopy [30,31]. The lacO array was inserted into chromosome 5 (line 5.1) or 13 (line 2.7) of HT1080 human fibrosarcoma cells that stably express lac repressor (lacI) fused to green fluorescent protein (GFP) to visualize the array position. The lacO integration sites are known to faithfully reflect the nuclear organization of their counterpart human chromosomes [32].

**Figure 1:**
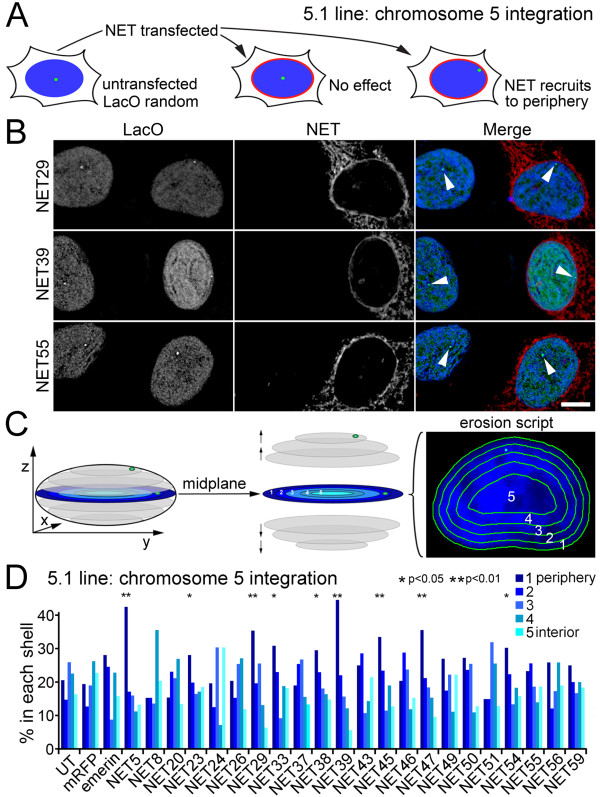
**A screen to identify NETs that recruit chromosomes to the nuclear periphery**. **(a) **An integrated lacO array marks chromosome 5 that tends to not be at the periphery in line 5.1. NETs fused to mRFP were exogenously expressed to screen for those involved in tethering chromatin to the NE. **(b) **Detection of the lacO array (arrowheads) and the transfected NETs (red) in the 5.1 line. The lacO array was not observed at the periphery in the untransfected cells (left) or the NET55 transfected cells, but was observed at the periphery in NET29 and NET39 transfected cells. Scale bar, 5 μm. **(c) **lacO array position was determined relative to five shells of equal area eroded from the periphery (1) to the centre (5) of the nucleus. To avoid errors in z from lacO arrays at the top or bottom of the nucleus, cells were only analyzed if the array occurred in the midplane where arrays occurring in the inner shells would be clearly internal. **(d) **Percentage of lacO-tagged loci in each of five erosion shells is plotted with the nuclear periphery (shell 1) to the left in dark blue and more internal localization occurring to the right and with lightening shades of blue. *n *= 50 cells for each NET. Single asterisks designate NETs with lower stringency *P*-values < 0.05 comparing the position of the array in the NET-transfected cells to the mRFP control using χ^2 ^tests; double asterisks designate NETs with higher stringency *P*-values < 0.01. Statistics for all NETs are given in Additional file 1. UT, untransfected.

Full-length NETs fused to monomeric red fluorescent protein (mRFP) were transiently expressed in line 5.1 (Figure 1a). This resulted in some accumulation of the fusion proteins in the endoplasmic reticulum (presumably due to saturation of binding sites at the NE), but a clear rim at the nuclear periphery was also observed (Figure 1b). Western blots for the mRFP tag revealed that the expressed NETs were not degraded (data not shown). The lacO array in line 5.1 is randomly distributed and tends to not be at the periphery [32] as can be observed in the untransfected cells in each image. While this minimal peripheral positioning was unchanged in cells expressing most NETs (for example, NET55 in Figure 1b), the array was often observed at the periphery in cells expressing certain NETs (for example, NET29 and NET39 in Figure 1b).

The position of the array with respect to the nuclear periphery was quantified in two dimensions to avoid errors from the unequal resolution between xy and z inherent to light microscopy. By using only cells where the lacO array could be visualized at the midplane (the focus point at which the nuclear diameter is at its widest; Figure 1c) such errors in z could be eliminated. As this would be expected to remove from consideration arrays at the top or bottom of the nucleus, it is likely to slightly underestimate array movement to the periphery. A script was then employed that erodes the total nuclear area, as defined by DAPI staining, into five concentric shells of equal area from the outside (shell 1) to the inside (shell 5) [3] (Figure 1c). The incidence of the array in each shell was quantified for each NET from at least 50 cells and the percentage of the total incidence in each sector of the nucleus plotted.

The line 5.1 lacO array is randomly distributed (not enriched at the periphery), with roughly 20% in each of the five shells, and expression of mRFP alone did not change this compared to untransfected cells (Figure 1d). Expression of NET5 (TMEM201), NET29 (TMEM120A), NET39 (PPAPDC3), NET45 (DAK) and NET47 (TM7SF2) all increased the incidence of the lacO array at the nuclear periphery with high statistical significance of *P *< 0.01 using χ^2 ^tests to compare the incidence of the array in the peripheral shell (shell 1) against the combined internal shells (shells 3, 4 and 5) between the NET-expressing cells and the mRFP-expressing cells. In contrast, emerin and most of the tested NETs had no strong significant effects on the position of the array, though NET23, NET33, NET38, and NET54 had minor effects using a lower stringency threshold of *P *< 0.05. Throughout the text high stringency (double asterisks in figures) indicates *P*-values < 0.01 while low stringency (single asterisks in figures) indicates *P*-values < 0.05; all *P*-values are given in Additional file 1.

**Figure 2:**
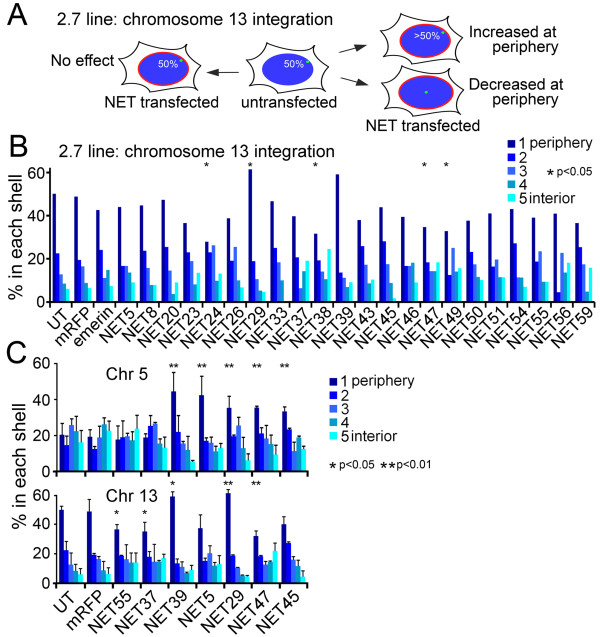
**NETs have distinct effects on a lacO array inserted into chromosome 13**. **(a) **In a different cell line the lacO array inserted in chromosome 13q tends to be at the periphery: so a NET could promote more peripheral localization or less. **(b) **Percentage of lacO-tagged 13q loci in each of five erosion shells is plotted with the nuclear periphery (shell 1) to the left in dark blue and more internal localization occurring to the right and with lightening shades of blue. *n *= 50 cells for each NET. **(c) **NETs that had strong effects on either cell line and two controls that did not (NET37 and NET55) were retested in two additional experiments to generate standard deviations and *P*-values. *P < 0.05 and **P < 0.01 comparing the position of the array in the NET-transfected cells to the mRFP control using χ^2 ^tests. Effects of individual NETs were highly repeatable with small error bars for standard deviation from the mean and *P*-values < 0.0001 for NET5, NET29, NET39, NET45 and NET47 in line 5.1. Statistics for each NET are given in Additional file 1. UT, untransfected.

As expected for an integration into the gene-poor chromosome 13 [2], the lacO array in line 2.7 tends to be close to the nuclear periphery (shell 1) in approximately 50% of cells (Figure 2a). NET29 and NET39 further increased this to approximately 60% (Figure 2b). Thus, their effect was qualitatively the same for both integration sites studied. In contrast, NET47, which had strongly promoted peripheral incidence for the array in line 5.1 (Figure 1d), did not further promote peripheral incidence of the array in line 2.7 (Figure 2b). In fact, in cells expressing NET47 the incidence of the array at the periphery was less than in the untransfected and mRFP controls.

The five NETs that most strongly influenced array position in line 5.1 (NET5, NET29, NET39, NET45 and NET47), the untransfected (UT) and mRFP controls, and two NETs that had no effect (NET37 and NET55) were subjected to additional rounds of transfection in both lines to confirm the effects and increase the statistical sample size (Figure 2c). All three experiments were highly reproducible, with NET29 and NET39 strongly promoting peripheral positioning in both lines with very small error bars for the standard deviation of the mean, while NET47 promoted peripheral positioning in line 5.1 but not in line 2.7. In line 5.1 analyzing the combined datasets further increased the statistical significance, with χ^2 ^tests yielding *P*-values of < 0.00001 for all NETs with effects on chromosome position when comparing the NETs to the mRFP-transfected controls (Additional file 1). In line 2.7 χ^2 ^tests revealed high statistical significance for NET29 and low statistical significance for NET39, with both increasing peripheral incidence of the array. In contrast, NET5 and NET45, though increasing peripheral incidence of the 5.1 line, had no significant effects on the 2.7 line. Interestingly, the reduced incidence of chromosome 13 at the periphery in cells expressing NET47 was found to be highly significant with a *P*-value of 0.002.

**Figure 3:**
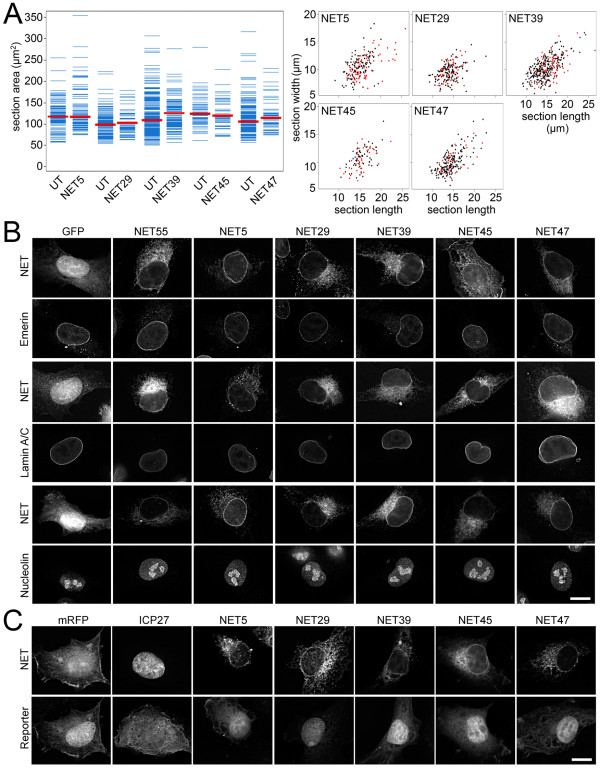
**Positioning effects are not due to changes in nuclear size and shape, distribution of other nuclear proteins, or loss of nuclear permeability**. HT1080 cells transiently transfected with tagged NETs were tested for parameters that could theoretically indirectly influence chromosome position. **(a) **The area of the nuclear midplane (left, distribution plots) and the longest and shortest distance across it (right, scatter plots) were measured and quantified for over 40 transfected cells for each NET. In each case the NET transfected cells were compared to untransfected (UT) cells in the same population. Only very moderate fluctuations in nuclear size or shape were observed. **(b) **Transfected cells were stained with antibodies for nucleolin to determine if NETs had indirect disruptive effects on nucleoli and for lamins and the NET emerin to determine if NETs generally altered NE organization. No changes in the distribution of these markers were observed. **(c) **The permeability barrier function of the NE in NET-transfected HT1080 cells was tested by expressing a reporter transport cargo in the same cells. The cargo contains two GFP molecules in tandem with a strong nuclear localization signal and a weak nuclear export signal so that it can shuttle but accumulates predominantly in the nucleus. If nucleocytoplasmic transport or NE permeability is compromised, the reporter accumulates more in the cytoplasm as when the herpesvirus protein ICP27, which interferes with nuclear transport through binding to Nup62, is co-expressed (second panel). None of the NETs affected the distribution of the reporter. For both (b) and (c) scale bar = 10 μm.

Gross changes in nuclear size and shape could in theory alter spatial genome organization [33], enabling more chromosomes to accumulate at the periphery. If a NET altered these parameters, it could have an indirect effect on chromosome positioning. Thus, nuclear size was determined by measuring the midplane area and nuclear shape was evaluated by measuring the longest and shortest cross-section in HT1080 cell nuclei expressing the NETs that affected chromosome position. Nuclear shape and size were largely unaffected by all these NETs (Figure 3a). The nucleolus could potentially also influence chromosome position but staining for nucleolin revealed no changes in the general appearance and number of nucleoli in cells transfected for the NETs that affected chromosome position (Figure 3b). The positioning effects were likely not due to disruption or redistribution of other NE proteins because lamin A/C, NPC proteins and the NETs emerin, SUN2 and nesprin 2 exhibited normal targeting in cells transfected with the NETs that affected chromosome position (Figure 3b and data not shown). The integrity of NPC function and the NE as a permeability barrier were also tested by investigating the distribution of a reporter transport cargo in cells co-transfected with the NETs that affected chromosome position. The transport cargo concentrated in the nucleoplasm in all NET-transfected cells while transfection with a positive control (ICP27) that has been shown to interfere with transport [34] caused the reporter cargo to accumulate in the cytoplasm (Figure 3c).

### NETs reposition specific chromosomes

**Figure 4:**
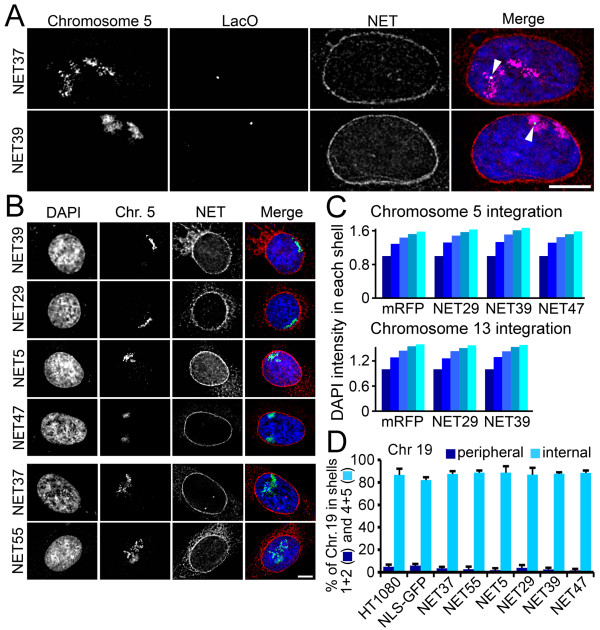
**NETs promote movement of whole chromosomes independently of the lacO array**. **(a) **NET-transfected cells subjected to FISH with whole chromosome paints and a probe for lacO. For NET39 both copies of chromosome 5 were in direct proximity to the NE, whereas the singly integrated array was merely closer; thus, relocalization to the periphery is independent of lacO sequences. **(b) **FISH with chromosome 5 paint on NET transfected HT1080 cells lacking a lacO array validated the lacO screen results. **(c) **Average DAPI fluorescence intensity in each nuclear erosion shell. No differences in DAPI distribution could be observed between the control cells and the NET transfected cells. *n *= 50 cells for each NET. **(d) **Quantification of chromosome 19 pixel intensity in the shells generated by the erosion macro. For the graph the percentage of the total intensity in the two outermost shells is summed (1+2, peripheral) and plotted against the summed percentage of the total intensity in the two innermost shells (4+5, internal). Error bars indicate standard deviation between the means of individual experiments. For both (a) and (b) scale bar = 5 μm.

To confirm that NET-induced repositioning of the lacO array faithfully represented the repositioning of the whole host chromosome, transfected 5.1 cells were subjected to fluorescence *in situ *hybridization (FISH) for the lacO array and for a chromosome 5 paint simultaneously. In the NET37-expressing control both the lacO array and the majority of chromosome 5 were internal whereas upon expression of NET39 the lacO array, the copy of chromosome 5 carrying the integrated lacO array, and the wild-type chromosome 5 were closer to the nuclear periphery (Figure 4a). Moreover, the lacO array itself was not directly at the periphery while other parts of the chromosome in images appeared to be in tight association with the NE. Importantly, this indicates that the lacO array itself does not contribute to NET39-mediated recruitment of chromosome 5 to the periphery.

To further test if chromosome repositioning effects are independent of the lacO array, parental HT1080 cells lacking the array were stably transfected with four NETs that had the strongest positioning effects in the first screen (NET5, NET29, NET39, and NET47) and controls (NLS[nuclear localization signal]-GFP, NET37 and NET55) that did not. FISH for chromosome 5 showed that the NETs that promoted peripheral repositioning of the lacO array integrated into chromosome 5 also repositioned the whole of chromosome 5 in parental HT1080 cells lacking the array (Figure 4b). NET37 and NET55 that failed to reposition the lacO array had no effect on chromosome 5 positioning in the parental HT1080 cells (Figure 4b).

The positioning effects were not due to a general increase in peripheral chromatin as no differences were observed in DAPI signal distribution within the shells between the mRFP control and NETs that recruited the array to the periphery (Figure 4c). Correspondingly, chromosome 19, which is normally located in the nuclear interior [3,35] remained in the nuclear interior. Indeed, approximately 90% of the hybridization signal from chromosome 19 was internally located in wild-type HT1080 cells and this distribution was unchanged in cells expressing each of the NETs that repositioned chromosome 5 to the periphery (Figure 4d). Thus, the NETs do not change the total distribution of chromatin in the nucleus, but must exhibit some specificity in their effects on chromosomes.

**Figure 5:**
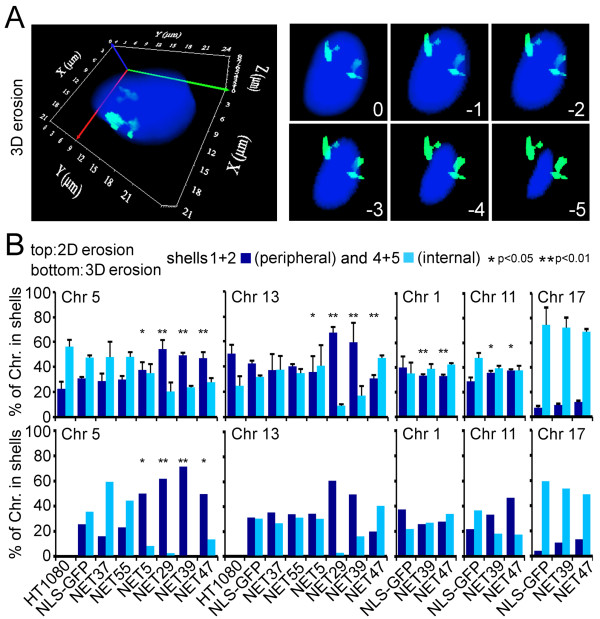
**NETs promote movement of partly overlapping and partly distinct sets of chromosomes**. **(a) **Similar to the two-dimensional analysis, an erosion approach was used to divide the three-dimensional nucleus (three-dimensional reconstructions) into shells of equal volume, but six shells instead of the five for the two-dimensional analysis were generated. **(b) **Quantification of whole chromosome painting in the absence of the array. Mean (± standard deviation) proportion (percentage) of the pixel intensity of human chromosomes 1, 5, 11, 13, and 17 in the two most peripheral and two most internal nuclear shells; *n *= 100 cells. The results from the three-dimensional analysis (lower panels) recapitulated those from the two-dimensional analysis (upper panels). *P < 0.05 and **P < 0.01, comparing the position of the chromosome in the NET-transfected cells to the NLS-GFP transfected control using Kolmogorov-Smirnov (KS) tests. Statistics for all chromosomes are given in Additional file 1.

To test for such specificity, suggested already by the different effects of some NETs for the two lacO lines, this investigation was extended to a larger set of chromosomes (1, 5, 11, 13 and 17) for a few NETs using both two-dimensional analysis of midplane images and three-dimensional shell erosion analysis (analysis from three-dimensional reconstructions). For the two-dimensional analysis the erosion shell macro was modified to measure the pixel intensity within each shell and the percentage of the total pixel intensity in the two most peripheral and two most internal shells was summed and plotted for 100 cells. For three-dimensional analysis a similar erosion script was applied to three-dimensional reconstructions from deconvolved stacks of images (Figure 5a). Because the large volumes for chromosome territories cross over several erosion shells, the unequal resolution in z should have little impact on three-dimensional measurements. For technical reasons the nucleus was divided into six concentric shells of equal area, rather than five as for the two-dimensional erosion analysis. As in the two-dimensional erosion analysis, the percentages of the total pixel intensity in the two outermost shells were combined and compared with the combined two innermost shells. As small regions of an internal chromosome can loop out to the periphery [36-38], the data are plotted as a percentage of the total pixel intensity so as to convey not just the movement of a chromosome region towards the periphery, but also how much of the whole chromosome moves towards the periphery. As the chromosome occupies multiple shells, statistical significance was assessed by comparing the distributions of chromosome intensities in the periphery (shells 1+2) between the NLS-GFP control and the NET by means of the Kolmogorov-Smirnov (KS) test.

Both the two- and three-dimensional analyses show the same tendencies that were observed using the lacO array as a marker for chromosome position. NET5, NET29, NET39 and NET47 promoted chromosome 5 positioning to the periphery and NET29 and NET39 further increased the incidence of chromosome 13 at the periphery. The effects of NET29, NET39, and NET47 were even more significant when analyzing chromosome 5 than the line 5.1 array (KS test *P*-values for two-dimensional analysis of 2.4 × 10^-10^, 6.3 × 10^-11^, and 4.1 × 10^-6^, respectively), and despite the low number of cells in the three-dimensional analysis (approximately 20), all had significant *P*-values (Additional file 1). NET5 was also significant in both two-dimensional and three-dimensional chromosome analysis, but went from highly significant with the lacO array to the lower significance cut-off of *P *< 0.05. This difference in the strength of the effect for NET5 likely reflects a smaller portion of the chromosome at the periphery, consistent with the images shown in Figure 4b. Chromosome 11 appeared visually to be moderately repositioned to the nuclear periphery by expression of NET39 and NET47 in both two-dimensional and three-dimensional analysis (Figure 5b); however, the *P*-values were only significant at the lower cut-off in the two-dimensional analysis. Chromosome 17, like chromosome 19 mentioned earlier, was unaffected by all NETs tested (Figure 5b; Additional file 1). Chromosome 1 did not increase in peripheral incidence with either NET, but instead appeared to exhibit a minor reduction in its peripheral incidence with both NETs. In all, NET39 promoted peripheral redistribution for three out of six chromosomes tested by the two-dimensional analysis while NET47 promoted peripheral redistribution for two of the chromosomes tested. Thus, NETs affect just a subset of chromosomes, with each NET promoting a particular pattern of chromosome repositioning.

### Chromosome repositioning effects of NETs are weakened without NE localization

**Figure 6:**
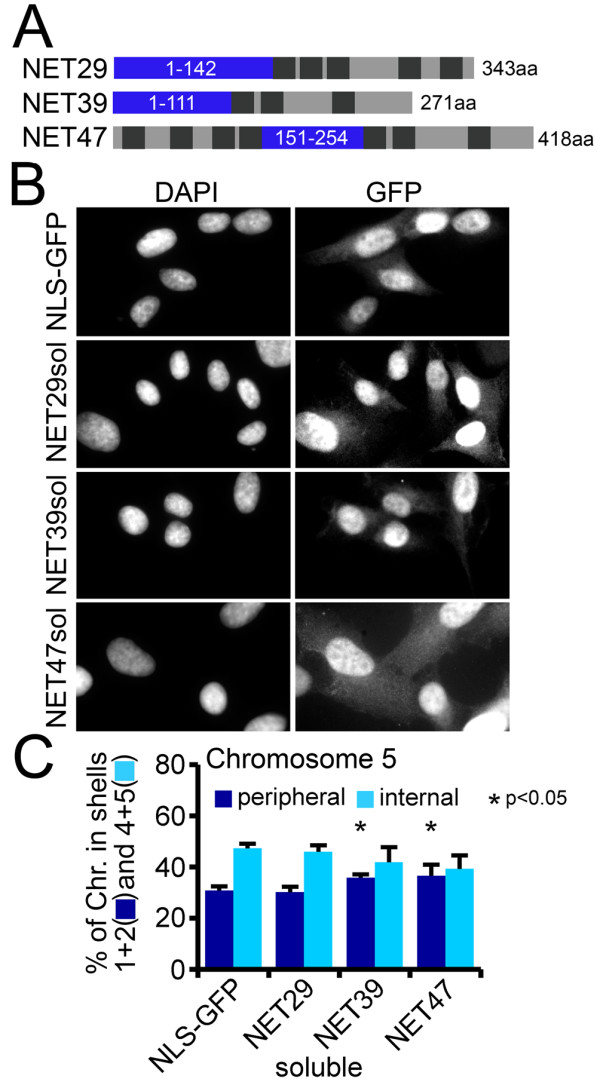
**Removal of the transmembrane segments reduces effects of NETs in chromosome repositioning**. Soluble fragments of NETs from their largest predicted nucleoplasmic segment were fused to an NLS and stably expressed in HT1080 cells to determine if they could influence chromosome repositioning. **(a) **Schematic diagram of each NET highlighting the soluble fragment in blue. The dark grey boxes indicate predicted transmembrane spans. **(b) **Images showing nucleoplasmic targeting of the GFP fusion constructs. **(c) **Cells expressing soluble fragments encoding the principal nucleoplasmic regions of NETs did not recapitulate the strong effects in repositioning observed with full-length NETs (Figure 5b). Error bars indicate standard deviation between the means of individual experiments. *P < 0.05 comparing the position of the chromosome in the NET-transfected cells to the NLS-GFP transfected control using KS tests. None of the soluble NET fragments yielded higher stringency *P*-values < 0.01. Statistics are given in Additional file 1.

To test if chromosome repositioning likely requires a membrane anchor on the NET, HT1080 lines were generated that stably expressed just the soluble nucleoplasmic regions of NETs (Figure 6a) fused to GFP and an NLS. Topologies were experimentally determined (data not shown) from accessibility in digitonin-permeabilized cells [39]. NET5 was not tested because it has two separate large nucleoplasmic regions. The fusions to the soluble nucleoplasmic regions of NET29, NET 39 and NET47 all accumulated diffusely in the nucleoplasm (Figure 6b), and two had weak effects (*P *< 0.05). However, none had the strong effects on chromosome positioning as were seen for their full-length counterparts (Figure 6c; compare with Figure 5b).

### NET-directed chromosome repositioning is reversible

**Figure 7:**
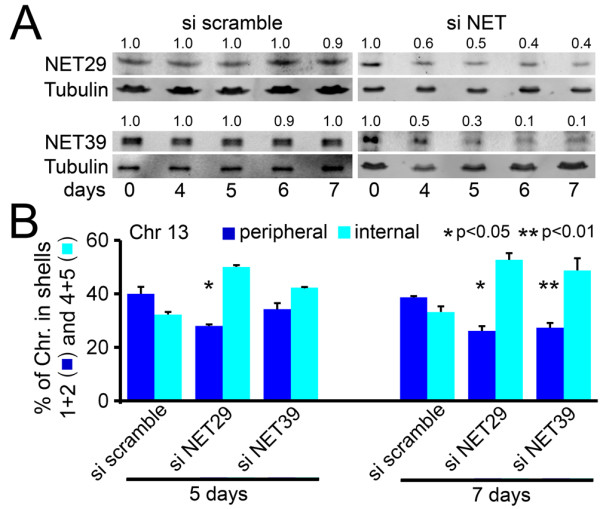
**Reversibility of chromosome repositioning effects**. **(a) **Wild-type HT1080 cells were separately treated with small interfering RNA (siRNA) oligos to deplete NET29 or NET39. Western blots of control siRNA and targeting siRNA-treated cells are shown. The signal intensity of bands on the blots was quantified and the band intensity for each siRNA was set to 1 for day 0. The normalized intensity of the bands over the time course is shown above each band. **(b) **Chromosome 13 position was quantified. Depletion of either NET reduced the endogenous peripheral positioning of chromosome 13. Error bars indicate standard deviation between the means of individual experiments. *P < 0.05 and **P < 0.01, comparing the position of the chromosome in the si NET cells to the si scramble control using KS tests. Statistics are given in Additional file 1.

Chromosome 13 tended to be peripheral in the HT1080 fibroblast cells and the peripheral incidence was increased with exogenous expression of NET29 or NET39. As the endogenous NET29 and NET39 proteins were also present in wild-type HT1080 cells, the tendency towards peripheral positioning for chromosome 13 could be due to this basal expression of the NETs. Thus, to test whether peripheral positioning of chromosomes by NETs is reversible, NET29 and NET39 were knocked down using RNA interference in wild-type HT1080 cells (Figure 7). Immunoblotting indicated that small interfering RNAs (siRNAs) reduced the protein expression levels to 30 to 50% of the normal endogenous levels by day 5 and 10 to 40% by day 7 (Figure 7a). Knockdown of each NET on its own was able to reduce the peripheral incidence of chromosome 13 by approximately 30% (Figure 7b). Both were statistically significant at day 7 of the knockdown using the KS test comparing to the scramble control from the same day of the knockdown (Additional file 1). This further confirms the NET function in positioning chromosomes as well as indicating that multiple NETs can act on the same chromosome.

### Restricted tissue expression of NETs that affect chromosome positioning

**Figure 8:**
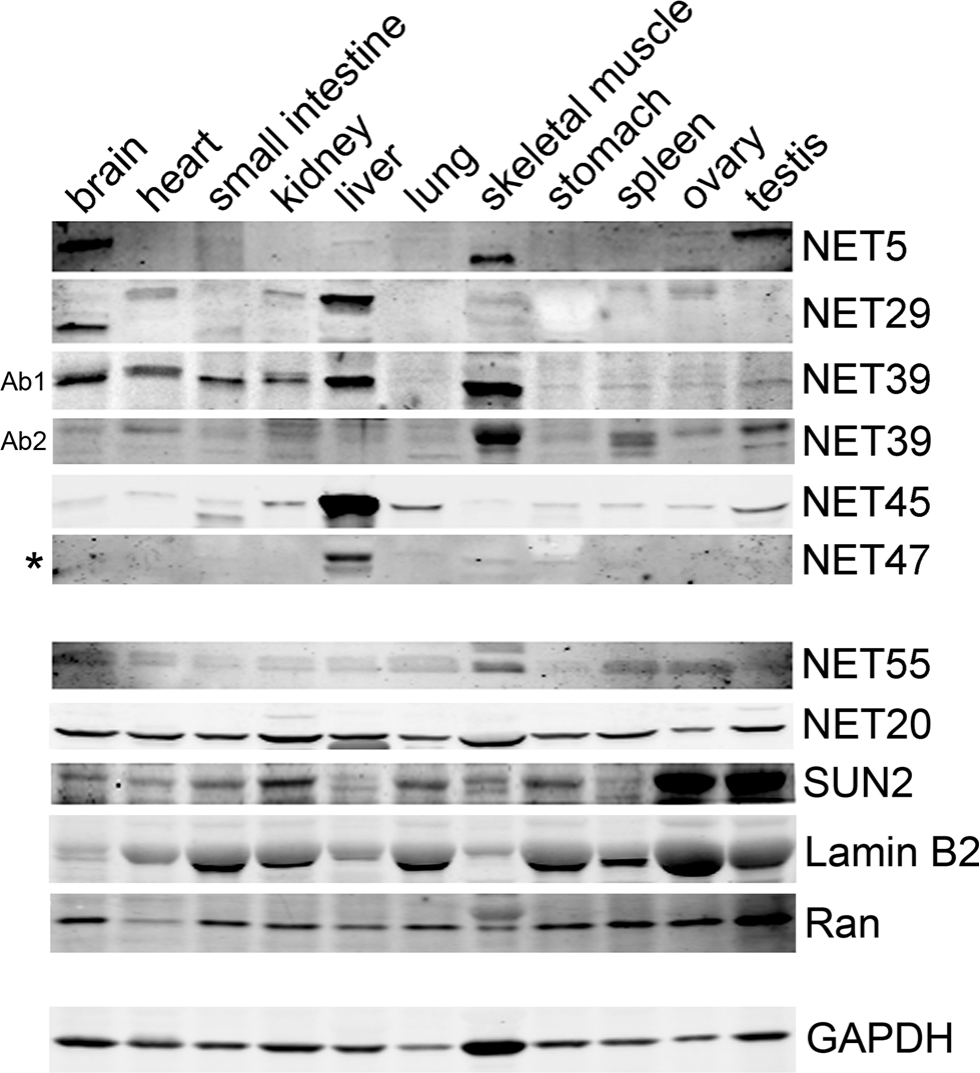
**NETs that recruit chromosomes to the nuclear periphery have restricted tissue expression**. Human tissue blots equally loaded for the tissues listed (20 μg in each lane) were probed with NET antibodies. NETs that had strong chromosome repositioning effects tended to be highly restricted in their expression. In contrast, several other NE proteins and controls were expressed in all tissues (lower panels). Ab1 and Ab2: two commercial antibodies to NET39 revealed restricted tissue expression and the highest levels of expression in muscle, but differed in the reactivity with some other tissues. Asterisk: NET47 migrates at 38 kDa instead of the calculated 46 kDa.

Given their effects on the positioning of certain chromosomes by both over-expressing and knocking down expression, these NETs could explain previous observations of tissue-specific chromosome positioning [11,12,40,41] if they are restricted in their expression. Thus, the protein levels of NETs with effects in the chromosome-repositioning screen were determined in different human tissues by western blot. All NETs that altered chromosome position were restricted in expression to a subset of tissues (Figure 8). As the NETs used in the initial screen were identified in a proteomic analysis of liver NEs [29] it is not surprising that NET45 and NET47 are very preferentially and highly expressed in liver. NET5 was expressed at very low levels in liver, but more strongly in brain, muscle and testis. Notably, the presence of bands with distinct molecular weights in some tissues indicates that some NETs have either splice variants or major post-translational modifications that are tissue-specific. In contrast, NET55 and NET20, which did not have repositioning effects, were widely expressed (Figure 8). Similarly, other NE-related proteins, SUN2, lamin B2, Ran, and the loading control GAPDH, were widely expressed in all tissues (Figure 8).

The protein levels on the western blot were measured in each tissue, summed, and the percentage of the total signal across all tissues observed in each particular tissue was calculated (Additional file 2). Thus, if a protein were expressed evenly across all 11 tissues it would have 9% of the total signal in each tissue. NET20, which visibly was relatively evenly expressed across all tissues, ranged from a low of 5% in ovary to a high of 13% in liver. In contrast, 77% of the total NET45 signal and 70% of the total NET47 signal was found in liver. Only 4% of the total NET45 signal and 3% of the total NET47 signal was found in kidney, making the relative expression in liver roughly 20-fold higher than in kidney for both NETs.

### NET involvement in chromosome positions in liver cells

**Figure 9:**
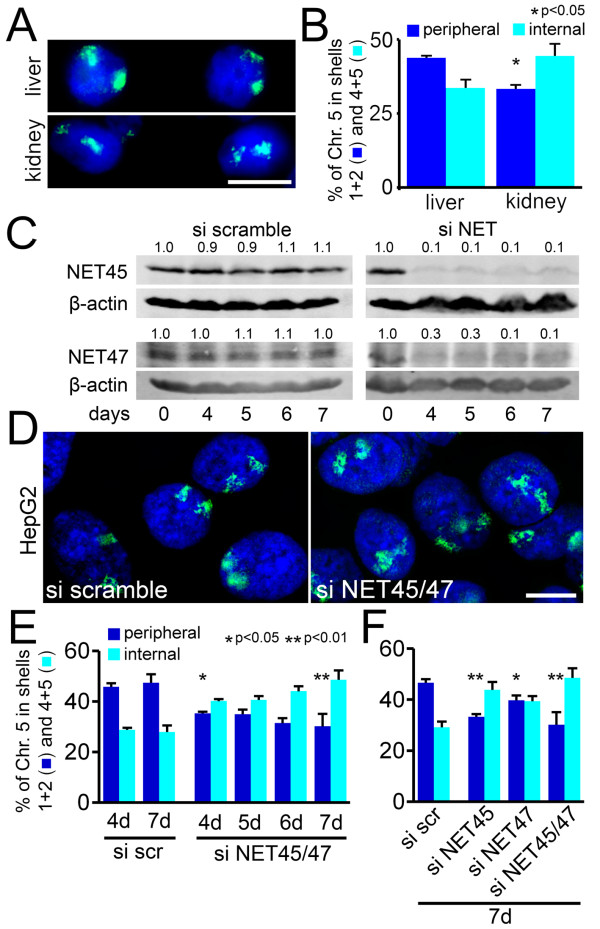
**Peripheral chromosome 5 position in liver is reduced by depletion of liver NETs**. **(a) **Chromosome 5 in human liver or kidney sections. Scale bar, 5 μm. **(b) **Chromosome 5 positioning was quantified for the two tissues (*n *= 100). **(c) **NET45 and NET47 were depleted by RNA interference in HepG2 cells, a liver-derived human cell line. A scrambled siRNA sequence was used as a control. The knockdown over time is shown by western blot. The signal intensity of bands on the blots was quantified and the band intensity for each siRNA was set to 1 for day 0. The normalized intensity of the bands over the time course is shown above each band. **(d) **Chromosome 5 in siRNA-treated HepG2 cells (left, scramble control; right, NET depleted). Scale bar, 5 μm. **(e) **Quantification of the results in (d); 100 cells were counted for each timepoint. The percentage of chromosomes relocated away from the periphery by the combined NET45/NET47 knockdown increased over time, also strengthening the statistical significance. **(f) **NET45 and NET47 were also tested individually to determine if both were needed for the normal peripheral localization. Each alone reduced the percentage of chromosome 5 at the periphery. The combined knockdown had a stronger effect than either NET alone (*n *= 100). Error bars indicate standard deviation between the means of individual experiments. *P < 0.05 and **P < 0.01, comparing the position of the chromosome in the si NET cells to the si scramble control using KS tests. Statistics are given in Additional file 1.

The highly preferential expression of NET45 and NET47 in liver together with their roughly 20-fold lower expression in kidney led us to test the position of chromosome 5 in human liver and kidney sections. Chromosome 5 was more associated with the nuclear periphery in human liver while being more associated with the nuclear interior in kidney with significant *P*-values from KS tests comparing the two tissues (Figure 9a, b).

HepG2 hepatocellular carcinoma cells retain many characteristics of hepatocytes [42], which comprise roughly 80% of liver. As in human liver sections, chromosome 5 was also predominantly peripheral in the HepG2 nuclei. To test whether this is dependent on the highly preferentially expressed liver NETs, NET45 and NET47 were co-depleted using siRNAs in HepG2 cells and the position of chromosome 5 monitored by FISH (Figure 9c, d). By day 4 protein levels had been depleted to between 10 and 30% of normal levels (Figure 9c). The percentage of chromosome 5 hybridization signal at the nuclear periphery decreased over time after combined siRNA treatment for the two NETs, but not for the control scrambled siRNA, and the proportion in the nuclear interior correspondingly increased (Figure 9e). At 4 days of depletion the chromosome repositioning was significant (*P *= 0.0169) using KS tests comparing the knockdown to the scramble control of the same day, and this improved to *P *= 7.6 × 10^-5 ^by 7 days. Depletion of each NET alone reduced the peripheral localization of chromosome 5 with statistical significance, but depletion of both NETs together reduced peripheral localization to only a slightly greater extent than NET45 alone (Figure 9f; Additional file 1). Thus, though both NET45 and NET47 contribute to the tethering of chromosome 5 in liver, it is not clear whether they act together or independently of one another.

## Discussion

We have identified nuclear membrane proteins that have extremely restricted patterns of expression in tissues and can promote particular patterns of spatial genome organization. Most NE-chromatin interactions described previously involved widely expressed proteins [20] mediating interactions with general heterochromatin [21-25,43]. Such interactions provide a mechanism for maintaining silent chromatin at the periphery that could help explain the partial relationship between chromosome positioning and gene density and indeed manipulating histone methylation can also affect heterochromatin positioning at the NE [44]. The release of chromosome 18 from the periphery due to lamin B1 defects [26] likely reflects such a mechanism because both lamin B1 and the core histones it binds [45-47] are ubiquitously expressed. The same should apply for the lamin A effects on chromosome position; however, lamin A mutations associated with disease affected gene and chromosome positioning in a more tissue-specific manner [28,48,49]. For example, lamin A mutations blocked release of a tissue-specific promoter from the periphery in the tissue matched to the promoter, but did not affect the peripheral localization in other tissues [48]. This suggests that lamin A is not the only protein involved in the initial tethering. Instead, we propose that lamins might reinforce a chromosome arrangement initially established by a tissue-restricted NET or be required for NE targeting of the NET, consistent with recent studies on lamin-associated domain organization by lamin B1 in conjunction with LAP2ß and HDAC3 [27] and generalized heterochromatin association with the periphery mediated by lamins in conjunction with the lamin B receptor (LBR) [43].

We postulate that the tissue-restricted NETs we have found function in similar complexes with lamins and chromatin proteins. There has been far more research to identify proteins on chromatin involved in tissue-specific patterns of genome organization compared to what has been done to identify the NE component. For example, in *Caenorhabditis. elegans*, release from the periphery of a muscle-specific promoter could be effected by the muscle-specific transcriptional regulator HLH-1 [10]. This argues that tissue-specific genes on chromosomes and transcriptional complexes sitting on them may be the mechanism by which specificity of chromosome positioning is conferred on the chromosome side because these tissue-specific genes would be unevenly distributed on the chromosomes. Different gene regulatory complexes that interact with different NETs could in theory work together to achieve a threshold affinity for chromosome tethering. That both NET29 and NET39 knockdown reduced the peripheral incidence of chromosome 13 in HT1080 cells and both liver-preferential NET47 and NET45 knockdown in the liver HepG2 cells reduced the peripheral incidence of chromosome 5 is consistent with this idea. However, much work still needs to be done to identify the specific binding partners on chromatin, to investigate the functions of these NETs in both matched and unmatched tissues, and test them in different combinations. Further, although the loss of chromosome repositioning function with the soluble fragments is consistent with the NETs being the physical tether at the NE, we cannot exclude the possibility that the NETs have a less direct function in modifying a different tether protein or chromatin component. Furthermore, although NET5, NET29, NET39 and NET47 were all resistant to a pre-fixation detergent extraction characteristic of lamina-associated NETs and characterized at the inner nuclear membrane by super resolution microscopy and in some cases immuno-electron microscopy [29-31], the fact that with overexpression much accumulates in the endoplasmic reticulum raises the possibility that some effects could be mediated indirectly from the cytoplasm.

The partial reduction in peripheral positioning observed for chromosome 13 in cells expressing NET47 could imply an indirect effect for these NETs in modifying a signaling pathway that can go in either direction, that is, movement towards the periphery or movement away from the periphery. However, it is also perfectly consistent with a model where different NETs recruiting partially overlapping and partially distinct sets of chromosomes to the periphery could indirectly cause the release of a particular chromosome from the periphery by increasing the peripheral tethering of a different chromosome.

Of the NETs that had effects in this screen, NET29, NET39, NET45 and NET47 are uncharacterized for any chromatin-related functions and there are no shared sequence characteristics among them. Only NET5 (also known as Tmem201 and Samp1) has been experimentally indicated to be able to bind to DNA or chromatin [50,51]. NET47 (also known as TM7SF2 and delta(14)-sterol reductase), which has a separate enzymatic function [52-54], may also be able to bind chromatin because it shares considerable sequence similarity with LBR [52,55] that binds heterochromatin protein 1 (HP1) [56]. Part of this HP1 binding site has been mapped close to the first transmembrane domain of LBR [57], a region that has high homology with NET47. Additionally, LBR has also been shown to be involved in spatial olfactory receptor gene organization [58]. Interestingly, both NET39 and NET47 have been implicated in tissue-specific processes, with NET39 playing a role in myogenesis [59,60] and NET47 knockout mice exhibiting a reduction in gene expression from a number of liver-specific genes [61].

Although there is still much work to do in testing whether these tissue-restricted NETs are tissue-specific in their functions and identifying the interaction partners/sites on chromosomes, the effects of NET47 on liver-specific gene expression [61] suggests that future work with these NETs will also answer questions on the functional consequences of particular patterns of spatial genome organization. Many aspects of the effects of spatial genome organization on gene expression remain contentious. For example, three studies tethering a lacO array to the NE yielded three different effects on gene expression [17-19]. Future studies manipulating tissue-restricted NETs in matched tissue systems may be the key to clearly answering many questions about the functional consequences of spatial genome organization.

## Conclusions

Certain chromosomes exhibit tissue-specific patterns of radial positioning in nuclei and several studies have demonstrated that chromosome tethering to the nuclear envelope can be achieved through an affinity mechanism. However, the endogenous proteins responsible for establishing a particular pattern of radial chromosome positioning have remained elusive. Here we identify several tissue-restricted nuclear envelope transmembrane proteins that can alter the radial position of chromosomes in the nucleus. Exogenous expression of each of these proteins in a general fibroblast cell line can recruit particular sets of chromosomes to the nuclear periphery and the peripheral distribution of certain chromosomes in differentiated cells can be reduced by depletion of these proteins. We predict that these newly discovered tissue-restricted NETs will likely play a pivotal role in dissecting the functional relevance of tissue-specific patterns of radial chromosome positioning in development.

## Materials and methods

### Plasmid construction

IMAGE clones for human NETs were inserted into a monomeric red fluorescent protein vector (pmRFP) as described [30]. Those used for stable cell lines were moved to the clontech pEGFP-N2 vector except for NET39, which was moved to pEGFP-C1 via EcoRI/BamHI sites. Soluble fragments are schematized in Figure 6. The reporter plasmid Rev_48-116_-GFP_2_-cNLS (NES-GFP_2_-cNLS) was described in [62].

### Cell culture and transfections

LacO cell lines were generated previously from HT1080 fibrosarcoma cells in the Bickmore laboratory [32]. Human HT1080 cells and these derivatives (lines 5.1, 2.7, and those stably expressing NETs) and human HepG2 hepatocellular carcinoma cells were maintained in high glucose DMEM supplemented with 10% fetal bovine serum, 100 μg/ml penicillin and 100 μg/ml streptomycin sulfate. Cells were plated at approximately 10% confluency to prevent their reaching confluency before fixation at 72 h post-transfection. DNA was transfected 12 h after plating using Fugene 6 (Promega, Madison, WI, USA) according to the manufacturer's instructions.

HT1080 cells were stably transfected using linearized plasmids carrying NET-GFP fusions. Transfectants were initially selected for with 500 μg/ml Geneticin for 2 weeks and surviving cells were further enriched for those expressing the GFP fusions by fluorescence-activated cell sorting (FACS). Cells were maintained thereafter with 100 μg/ml Geneticin.

### Antibodies and tissue western blots

Human tissue blots (IMB-103, IMGENEX, San Diego, CA, USA) were probed using standard procedures with antibodies to NETs. Antibodies against NET5 (06-1013), NET29 (06-1018), NET55 (06-1029), SUN2 (06-1038) were all rabbit polyclonals generated to peptides from Millipore (Temecula, CA, USA). Antibodies to NET39 were rabbit polyclonals from either Millipore (06-1025; Ab1) or Proteintech (20635-1-AP [Chicago, IL, USA]; Ab2). Antibodies to NET47 were rabbit polyclonals from either Millipore (06-1026) or Professor Rita Roberti, Perugia University [53]. NET45, mouse monoclonal antibodies were generated against a recombinant GST-NET45 fusion protein lacking transmembrane domains using methods previously described [63]. NET20 antibodies were rabbit polyclonals made to peptide KFKRNLSVEAEVDLLSYCAR (amino acids 83 to 102). Lamin B2 rabbit polyclonals have been previously described (3932) [64]. Ran mouse monoclonal and GAPDH rabbit polyclonal antibodies were from Beckton-Dickenson (610341, San Jose, CA, USA) and EnoGene (E1C604, New York, NY, USA), respectively. Protein bands were visualized with IR680- or IR800-conjugated secondary antibodies using a LI-COR Odyssey (LI-COR, Lincoln, NE, USA). Quantification of bands in western blots was performed using the LI-COR Odyssey Application Software version 3.0. Integrated intensities were calculated from an equal size box drawn around each band in each tissue (Table S2a in Additional file 2). To assess the variation of protein abundance among tissues, the intensities were summed within each protein for all tissues, and then expressed as a percentage of the signal per tissue (Table S2b in Additional file 2).

For other western blots, protein lysates from knockdown experiments were separated on SDS-PAGE and transferred to membranes that were probed with the primary antibodies listed above and as loading controls anti-actin (mouse monoclonal; A1978, Sigma, St. Louis, MO, USA) or anti-tubulin (mouse monoclonal; T6074, Sigma). Because GFP is denatured during FISH procedures, to visualize GFP-NETs antibody A11122 (Life Technologies, Grand Island, NY, USA) against GFP was used. Antibodies against lamin A/C (rabbit polyclonal 3262) [64], emerin (MANEM1 5D10) [65], nucleolin (rabbit polyclonal Ab22758, Abcam, Cambridge, UK) were also used to stain the NET-transfected cells in Figure 3.

### Immunofluorescence staining and permeability assays

Transiently transfected cells were fixed in 4% paraformaldehyde for 7 minutes, then permeabilized in 0.2% Triton X-100/PBS for 5 minutes and blocked with 2% BSA in PBS. They were then incubated with primary antibodies at 37°C for 1 h in the same 2% BSA solution, washed three times in PBS and then incubated with secondary antibodies for 45 minutes. After washing in PBS, cells were mounted in fluoromount G (EM Sciences, Hatfield, PA, USA) and analyzed by microscopy.

For permeability assays HT1080 cells were transiently transfected with tagged NETs fused to mRFP and the transport reporter NES-GFP_2_-cNLS [62], which has both nuclear export and import signals so that it shuttles, but the import signal is dominant. Thus, the reporter should accumulate predominantly in the nucleoplasm unless transport or NE permeability is compromised. As a positive control for disruption of transport, cells were co-transfected with the reporter and pmRFP-C1 plasmid encoding WT ICP27. After 24 h cells were fixed and processed for microscopy.

### siRNA knockdown of NETs

siRNA oligos were used for knockdown of NET29 (5'- CUA AGU UUG CCU ACA AGG A[dT][dT]-3' and 5'- UCC UUG UAG GCA AAC UUA G[dT][dT]-3') and NET39 (5'- CUA CCU CAC CAU GGA CAU CUA[dT][dT]-3' and 5'- UAG AUG UCC AUG GUG AGG UAG[dT][dT]-3') in HT1080 cells; 8 μg of either was transfected into 10^6 ^cells using a Nucleofector (Lonza, Cologne, Germany) with program L-005 and solution T. The control siRNA was a scrambled sequence. Knockdown was confirmed using the Millipore NET29 and Proteintech NET39 antibodies.

For knockdown of NET45 and NET47 in HepG2 cells, 3 μg each of Smartpools (Thermo Fisher Scientific, Waltham, MA, USA: NET45 L-006808-00-0020, NET47 L-005744-00-0020) alone or in combination were transfected into 1.5 × 10^6 ^cells using nucleofection (Lonza, solution V, program T-028). The control siRNA was a scrambled sequence. Knockdown was confirmed using the monoclonal NET45 antibody and the NET47 antibody from Professor Rita Roberti.

### Fluorescence *in situ *hybridization

For immuno-FISH, cells were fixed in 4% paraformaldehyde, aged 2 days, then permeabilized in 0.2% Triton X-100/PBS for 5 minutes and incubated with primary antibodies at 37°C for 1 h and secondary antibodies for 45 minutes. After washing in PBS, cells were again fixed with 2% formaldehyde for 5 minutes to fix antibodies prior to denaturing FISH steps. Cells were permeabilized again with 0.5% Triton X-100, washed in PBS and then pre-equilibrated in 2× SSC and treated with RNase (100 μg/ml) at 37°C for 1 h. After washing in 2× SSC, cells were dehydrated with a 70%, 90% and 100% ethanol series. Slides were heated at 70°C and then submerged in pre-heated (80°C) 70% formamide/2× SSC (pH 7.2) for 20 minutes followed by another ethanol dehydration series. Slides were air-dried and hybridized to biotin-labeled chromosome paints (CamBio, Cambridge, UK) or a lacO probe labeled with digoxigenin. Hybridizations were incubated for 2 days at 37°C, then washed in 2× SSC at 45°C followed by 0.1× SSC at 60°C. Slides were then pre-equilibrated in 4× SSC, 0.1% Tween-20 and blocked with BSA for 15 minutes before incubating with avidin or digoxigenin antibodies (Roche). DNA was visualized with DAPI (4,6-diamidino-2 phenylindole, dihydrochloride) and coverslips mounted in fluoromount G (EM Sciences, Hatfield, PA, USA).

Human tissue sections were obtained according to local ethics protocols, formalin fixed, paraffin embedded and sectioned at 5 to 6 μm thickness onto slides. Sections were subsequently prepared for whole chromosome painting according to published protocols [66]. Briefly, sections were deparaffinized in 100% xylene at 45°C, rehydrated with an ethanol series, permeabilized with 1 M Na isothiocyanate at 80°C for 30 minutes followed by 50 μg/ml pepsin (Sigma P-6887) in 0.01 N HCl at 37°C for 45 minutes. Cells were then dehydrated with an ethanol series and whole chromosome painting was performed as above.

### Microscopy

Most images were obtained using a Nikon TE-2000 microscope equipped with a 1.45 NA 100× objective, Sedat quad filter set, PIFOC Z-axis focus drive (Physik Instruments, Cranfield, UK), and CoolSnapHQ High Speed Monochrome CCD camera (Photometrics, Marlow, UK) run by Metamorph image acquisition software. Image stacks (0.2 μm steps) were deconvolved using AutoquantX (Media Cybernetics, UK). Micrographs were saved from source programs as 12-bit.tif files and analyzed with Image Pro Plus software and/or prepared for figures using Photoshop 8.0.

The positional distribution of the lacO array was determined using a macro (available on request) written in Visual Basic within Image Pro Plus. In brief, the total nuclear area was automatically measured on DAPI images, then divided into five shells of equal area through eroding 20% of total area from the outer limits of the DAPI-defined nucleus. The nuclear shell containing the lacO spot was determined, exported to Microsoft Excel and summed for each cell. The same shells were also applied for DAPI or chromosome intensity measurements where a manually intensity-thresholded and background subtracted image of the chromosomes was used (two-dimensional shell erosion macro).

To quantify the three-dimensional position of FISH-labeled chromosomes within the nucleus, a macro (three-dimensional shell erosion macro) was devised within Image Pro Plus 7.0 software based on a similar erosion script to that used for the two-dimensional analysis. Prior to running the macro, images of Alexa 488-labeled chromosomes and DAPI-labeled nuclei were deconvolved using Autoquant X, exported into Image Pro Plus 7.0, and saved as two separate files.

Within the macro each z level of the nucleus was automatically thresholded and converted into a three-dimensional binary image so that weaker DAPI stained regions such as nucleoli would not be interpreted as holes for nuclear area when applying the erosion script. The chromosomes were manually thresholded and then converted into a three-dimensional binary image. This binary image required a minimum density so that background spots would be eliminated. Though this had the disadvantage that some control cells with very wide and diffuse chromosome distributions in the nuclear interior had reduced internal chromosome measurements, nonetheless, the only effect on data interpretation was to lessen differences between the controls and NETs that repositioned chromosomes. A three-dimensional distance filter was applied to the three-dimensional nucleus image to allow erosion of the nucleus based on distance from the center. Three-dimensional binary images were produced from the distance image by dividing the total area by 6 and then eroding the area by 1/6th each time from the preceding image to produce six images.

The binary chromosome image was then combined with each of the six eroding nucleus images and the degree of co-localization was then measured in each combined image and the percentage of chromosome in each eroded three-dimensional nuclear area calculated. Subtraction of the percentage in sequential shells then gave the percentage of the chromosome in each unique shell.

To quantify nuclear size, the area of the midplane was measured using Image Pro Plus 7.0 and plotted as distribution plots. For nuclear shape the longest and shortest distances across the midplane images were also measured automatically with the built-in functions of the software and plotted as scatter plots.

### Bioinformatics analysis and statistics

Repositioning of lacO arrays was assessed by comparing the number of arrays present within the outermost shell (shell 1) against the internal area (combined shells 3+4+5) between each NET and the mRFP control by means of the one-tailed chi-squared test with a significance threshold of *P *< 0.01 for high stringency or *P *< 0.05 for low stringency.

Chromosomes occupy a large area, compared to the lacO arrays, spanning several shells. For this reason, the chromosome data were calculated as the percentage of the total chromosome signal intensity that was contained within each shell. Chromosome repositioning was assessed by comparing the distribution of chromosome intensities within the periphery (shells 1 and 2) between each NET and the NLS-GFP, scramble siRNA and NET siRNA or liver and kidney by means of the KS test with a significance threshold of *P *< 0.01 for high stringency and *P *< 0.05 for low stringency.

## Abbreviations

BSA: bovine serum albumin; FISH: fluorescence *in situ *hybridization; GFP: green fluorescent protein; KS: Kolmogorov-Smirnov; lacO: lac operator; LBR: lamin B receptor; mRFP: monomeric red fluorescent protein; NE: nuclear envelope; NET: nuclear envelope transmembrane protein; NLS: nuclear localization signal; PBS: phosphate-buffered saline; siRNA: small interfering RNA.

## Authors' contributions

NZ designed experiments, performed most of the experimentation and data analysis, and helped draft the manuscript. SB trained NZ in FISH techniques and helped with human tissue section staining. DAK wrote the chromosome repositioning analysis algorithms and helped with data analysis. JIH helped with data analysis. VL did preliminary work on the lacO array repositioning screen. NK helped to immunostain human tissue blots. DGB helped with protein quantification. KNR and GEM generated the NET45 antibody. DJH provided human tissue sections. WAB provided reagents and helped write the manuscript. ECS designed the study and wrote the manuscript. All authors read and approved the final version of the manuscript.

## Supplementary Material

Additional file 1 **Table S1 - statistics for Figures **1, 2, 5, 6, 7, **and **9. Repositioning of lacO arrays (for Figures 1d and 2b, c) was assessed by comparing the number of arrays present within the outermost shell (shell 1) against the internal area (combined shells 3+4+5) between each NET and the mRFP control by means of the one-tailed chi-squared test. Chromosome repositioning was assessed by comparing the distribution of chromosome intensities within the periphery (combined shells 1 and 2) between each NET and the NLS-GFP (Figures 5b and 6c), scramble siRNA and NET siRNA (Figures 7b and 9e, f) or liver and kidney (Figure 9b) by means of the KS test. *P*-values for each sample are listed with a significance threshold of *P *< 0.01 for high stringency (red) or *P *< 0.05 for low stringency (blue). Click here for file

Additional file 2 **Table S2**. Quantification of bands in western blots was performed using the LI-COR Odyssey Application Software version 3.0. Integrated intensities were calculated from an equal size box drawn around each band in each tissue (Table S2a). To assess the variation of protein abundance among tissues, the intensities were summed within each protein for all tissues, and then expressed as a percentage of the signal per tissue (Table S2b). Click here for file
